# Evaluation of Whatman FTA cards for the preservation of yellow fever virus RNA for use in molecular diagnostics

**DOI:** 10.1371/journal.pntd.0010487

**Published:** 2022-06-15

**Authors:** Emily H. Davis, Jason O. Velez, Brandy J. Russell, A. Jane Basile, Aaron C. Brault, Holly R. Hughes

**Affiliations:** Centers for Disease Control and Prevention, Division of Vector-Borne Diseases, Fort Collins, Colorado, United States of America; Universidade do Estado do Para: Universidade do Estado do Para, BRAZIL

## Abstract

Yellow fever virus (YFV) is a flavivirus that frequently causes outbreaks of hemorrhagic fever in Africa and South America and is considered a reemerging public health threat. Accurate diagnosis of yellow fever (YF) disease is critical as one confirmed case constitutes an outbreak and may trigger a mass vaccination campaign. Highly sensitive and specific molecular diagnostics have been developed; however, these assays require maintenance of cold-chain during transport of specimens to prevent the degradation of viral RNA prior to testing. Such cold-chain requirements are difficult to meet in some regions. In this study, we investigated Whatman FTA cards as an alternative stabilization method of YFV RNA for use in molecular diagnosis. Using contrived specimens, linear regression analysis showed that RNA detection from a single 6mm FTA card punch was significantly less sensitive than traditional RNA extraction; however, pooling RNA extracted from two FTA punches significantly lowered the limit of detection to be equal to that of the traditional RNA extraction gold standard. In experiments addressing the ability of FTA card methodology to stabilize YFV RNA at variable temperature, RNA could be detected for more than two weeks following storage at 25°C. Even more promising, YFV RNA was detectable on cards held at 37°C from two days to over two weeks depending on viral input. FTA cards were also shown to stabilize YFV RNA at high humidity if cards were desiccated prior to inoculation. These results support that FTA cards could be cost effective and easy to use in molecular diagnosis of YF, preserving viral RNA to allow for positive diagnoses in situations where maintaining cold-chain is not feasible.

## Author summary

Yellow fever virus (YFV) is the etiological agent of yellow fever (YF), a hemorrhagic disease endemic to sub-Saharan Africa and tropical regions of South America. Due to the threat YF poses to public health, a single case of constitutes an outbreak, making accurate diagnosis paramount. Viremia during YFV infection is variable and may be quite low depending on disease severity and the timing of sample collection. In addition, viral RNA is highly liable, especially in the hot and humid conditions that accompany YFV transmission season. To reduce RNA degradation, clinical samples are transported to diagnostic laboratories via cold chain. As cold chain is not always reliable or available, the capacity of certain regions to perform the molecular diagnosis of yellow fever is stunted, leading to a delay in diagnosis and outbreak management. Here we optimized a protocol for the incorporation of Whatman FTA cards into the YF molecular diagnostic with the goal of producing a cold chain-independent viral RNA stabilization method. These cards are compact, easy to use and were shown to inactivate YFV and stabilize YFV RNA at high temperature and humidity. The implementation of this protocol in endemic regions could aid in the rapid and accurate diagnosis of YF.

## Introduction

Yellow fever virus (YFV) is a flavivirus endemic to 47 countries in sub-Saharan Africa, Central America, and South America. The virus is responsible for periodic outbreaks of yellow fever (YF) disease, a hemorrhagic disease with a case fatality rate of 30–60% in severe cases [1]. Each year there is an estimated 200,000 cases of YF and 30,000 YF-associated deaths [2]. As the majority of YF cases are asymptomatic, the prevalence of YFV is considered to be much higher than annual incidence rates suggest. Thus, the WHO classifies a single confirmed case of YF as an outbreak, requiring immediate ring vaccination with the live-attenuated vaccine strain, 17D [3]. Although vaccination against YF is part of routine vaccination programs in many YF endemic countries, there has been an emergence of YFV in urban areas with large, unvaccinated populations. The increased risk of YF outbreaks and limited YF vaccine supplies underscores the need for accurate YFV diagnostics. False negatives could lead to larger outbreaks and false positives could lead to unplanned vaccine usage.

Diagnosing YF can be difficult as patients present with non-specific, flu-like symptoms that can be confused with leptospirosis, malaria, and other viral hemorrhagic fevers during differential diagnosis. As such, laboratory diagnosis is required to confirm YF. Both serological methods (YFV IgM or IgG assays followed by plaque-reduction neutralization tests) and/or detection of YFV-specific RNA are included in the WHO algorithm for laboratory diagnosis of YF [4]. Although these assays are successfully used to accurately detect YF, there are important caveats that must be addressed. Serological diagnosis of YF is complicated by the well characterized flavivirus cross-reactive antibody response [5]. Infection with other flaviviruses such as dengue (1–4), Zika, and West Nile viruses must be ruled out by additional serological testing and confirmed at regional reference laboratories. Additionally, IgM generated in response to a wild-type (WT) YFV infection is indistinguishable from IgM generated after vaccination, including in rare cases of vaccine-associated viscerotropic disease, making serological diagnosis during mass vaccination campaigns difficult [6].

Although YF molecular diagnostic assays can differentiate between WT and rare adverse events associated with 17D vaccination [7,8], samples are required to be transported on wet ice if arriving within one day and at -20°C if arriving after more than one day. This cold-chain is required to prevent the degradation of YFV RNA but is often not realistic in YF-endemic regions where transmission season is accompanied by hot and humid weather [3]. Furthermore, viremia during YFV infection is transient and highly dependent on when the sample is collected, resulting in some YF clinical samples having very low levels of viral RNA. The use of serological or molecular methods for YF diagnosis is determined by the timing of specimen collection (i.e., acute vs. non-acute) and laboratory capacity of the region. South American countries routinely use the ‘YFall” primers for molecular diagnosis [9] while African countries prefer plaque reduction neutralization tests (PRNTs) and the CDC Arboviral MAC ELISA for serological testing. With the recent WHO evaluation of a qRT-PCR kit for the molecular diagnosis of YFV [10], it is likely that molecular diagnosis will become more routine in Africa, underscoring the need for RNA stabilization as a critical factor for the implementation of YF molecular diagnostics in new regions.

Whatman FTA cards have been shown to be effective in the molecular diagnosis of multiple viral and bacterial diseases [11–24]. The current WHO diagnostic algorithm calls for the molecular diagnosis of YF from serum samples, which are collected at clinics and diagnosis is completed at regional reference labs [25]. Herein, we show how incorporating FTA cards into YFV molecular diagnostics has the potential to improve YFV surveillance in remote, endemic regions.

## Materials/Methods

### Viruses

The vaccine sub-strain 17D-204 [titer: 7 log_10_ plaque forming units (pfu)/ml] and WT strain Asibi (titer: 6 log_10_ pfu/mL) used in this study were obtained from the CDC Arboviral Disease Branch, Arbovirus Reference Collection. Experiments involving 17D-204 virus were performed according to biosafety level 2 requirements and experiments involving Asibi virus were performed according to biosafety level 3 safety requirements. Serial dilution of virus was performed in PBS (Gibco, Massachusetts, USA) or flavivirus-negative human serum (EMD Millipore, Massachusetts, USA).

### FTA card protocol, RNA extraction and genome amplification

Contrived specimens were spotted onto FTA micro cards (WHAWB120205, Sigma-Aldrich, St. Louis, USA), allowed to dry and RNA extracted from discs punched from the card according to the manufacturers protocol (i.e., 140 μL of sample distributed evenly over the sample area). In order to minimize cross-contamination, cards were also pre-punched with a sterilized, 6 mm hole punch prior to inoculation. Punches were inoculated with 10 μL of contrived specimen and allowed to dry for one hour at room temperature in the biosafety cabinet. Individual punches were then placed into 1.5 mL tubes and stored as described until RNA extraction was performed using the QIAmp viral RNA extraction kit (Qiagen, California, USA).

To extract RNA, individual FTA punches were placed directly into 140 μL of PBS, thoroughly vortexed, added to 560 μL buffer AVL and allowed to incubate for 10 minutes at room temperature. When pooling RNA from two punches, individual punches were placed into 70 μL of PBS and vortexed. The extracts in PBS were then combined (i.e., 140 μL total) and added to 560 μL of AVL. The remaining steps of the QIAmp protocol were then followed as previously described [23]. Final elution was performed using 60 μL of Buffer AVE. RNA from the same preparation of YFV spiked serum was simultaneously extracted according to the manufacturer’s protocol (i.e., sample placed directly into buffer AVL).

Viral RNA was detected using the Quantitect probe RT-PCR kit (Qiagen, California, USA) with YFall primers as previously described [9] in a total reaction volume of 25 μL. All samples were tested in triplicate, using 10 μL of RNA. While using high input elute may result in inclusion of more inhibitors in the reaction, inhibition was not observed in our limited analysis using normal human serum.

### Inactivation of YFV after FTA inoculation

Confirmation of viral inactivation was performed using institutionally approved protocols. The vaccine strain, 17D-204 virus was used as a surrogate for all YFVs in order to reduce biosafety concerns. FTA punches were inoculated with 10 μl of 17D-204 virus (5 log_10_ pfu/ml), allowed to dry, and were rehydrated in 100 μL sterile water. The resulting FTA extract was used in two assays to confirm inactivation. First, 100 μL of FTA extract was inoculated onto Vero cells and allowed to incubate for 1 hour at room temperature after which DMEM media was added, and the flask placed at 37°C/5% CO_2_ and allowed to incubate for one week. Cells were monitored for cytopathic effect (CPE) which occurs with vaccine strains of YFV. After one week, 100 μL of media from the week one flask was transferred onto fresh Vero cells. This process was repeated for three weeks. Secondly, the FTA extract was used in a viral plaque assay. Ten-fold serial dilutions (1:10–1:1,000,000) of FTA extract were prepared in Bovine albumin-1 media (BA-1) and used to perform a plaque assay as previously described [26].

### Role of environmental conditions on RNA stability

For stability experiments, 17D-204 was diluted in PBS or human, flavivirus-negative serum and inoculated onto punches at concentrations of 7 log_10_ pfu/mL (100,000 pfu/punch), 6 log_10_ pfu/mL (10,000 pfu/punch), 5 log_10_ pfu/mL (1,000 pfu/punch), 4 log_10_ pfu/mL (100 pfu/punch), 3 log_10_ pfu/mL (10 pfu/punch) and 2 log_10_ pfu/mL (1 pfu/punch). The ability of FTA cards to stabilize YFV RNA at an elevated temperature was measured by holding FTA punches with variable titers of YFV at 37°C for two weeks and comparing RNA positivity to control punches held at room temperature (~25°C) for the same amount of time. Samples were testing in triplicate and experiments were conducted in duplicate, leading to n = 6 for all groups. Punches in the 37°C group were placed directly into heat blocks after inoculation to ensure that any effect of temperature on the drying process was captured in the experiment. In experiments utilizing a single punch, RNA was extracted from one punch daily for seven days and at 14 days post-inoculation. For studies using pooled RNA from two punches, only 10 pfu/punch and 1 pfu/punch were utilized. Once a punch no longer tested positive for YFV RNA, punches from that dilution were removed from collection on subsequent days.

Laboratories in YF-endemic areas are often subject to high humidity. To assay the effect of humidity on the utility of the FTA cards, a two-part experiment was conducted using the one punch protocol described above. The first part sought to determine whether exposure of cards to high humidity post inoculation affected their ability to stabilize YF RNA. FTA cards were inoculated with 10 pfu/punch or 1 pfu/punch of 17–204 virus and incubated at high humidity (80–85%). RNA was extracted from one punch daily for seven days. Humidity readings were taken at each time point using a humidity and temperature pen (Fisher Brand) to ensure no large fluctuations in humidity occurred due to opening and closing of the incubator door. The second evaluation sought to determine whether exposure of cards to high humidity prior to inoculation affected their ability to stabilize YF RNA. FTA cards were incubated at 37°C and high humidity (80–85%) for one, two, and three days prior to inoculation cards were removed from the incubator, immediately punched, and inoculated with 10 pfu/mL or 1 pfu/mL of 17–204 virus. Punches were returned immediately to the incubator so that drying would occur at high humidity.

Finally, the effect of desiccating cards that were previously exposed to humid conditions was assayed to establish if dry FTA cards were required to stabilize RNA after inoculation. FTA cards were placed in an incubator at 37°C for three days at 75–80% humidity, then were transferred into plastic bags containing one or two silica 1-gram gel desiccation packets (Dry & dry, California, USA) and sealed. A card placed in a bag with no desiccation packets was also included as a control at each timepoint. Bags were returned to the incubator for one, two or seven days prior to inoculation when FTA cards were punched and inoculated with 10 pfu/punch or 1 pfu/punch of 17D-204 virus. The punches were returned immediately to the bag containing the specified number of desiccant packets and placed into the incubator. RNA was extracted from punches and assayed for YFV RNA positivity every day for seven days. The experiment was repeated with a different brand of desiccation packet (Whatman FTA, 1-gram packets) to compare the efficacy of 1-gram desiccation packets.

### Statistics

Limit of detection (LOD) and time to negative result were calculated by linear regression modeling, estimating when the mean C_t_ value reached the cut-off threshold of 37. The goodness of fit for these regressions was calculated using R^2^ values. For high titer samples, the mean C_t_ value never reached 37 before the end of the time course, so the time to detection loss was extrapolated based on the linear regression model. Significant differences in LOD and time to negative result were determined by calculating the likelihood that the 95% confidence intervals of C_t_ value 37 for two experiments (95% CI) would overlap as described previously [27]. In cases where the time to negative result was estimated due to limitations in data, a comparison of 95% CIs was not completed as the mean number of days to a C_t_ of 37 was extrapolated.

## Results

### Inoculation of YFV onto FTA cards results in viral inactivation

The vaccine strain of YFV, 17D-204, was confirmed to be inactivated after storage on FTA cards. No CPE was observed in Vero cells inoculated with 17D-204 FTA punch extract after three blind passages of 1 week each. Additionally, no plaques were observed after a plaque assay was performed on 17D-204 FTA punch extract.

### RNA from vaccine and WT strains of YFV can be detected after inoculation onto FTA cards

To determine if vaccine and WT strains of YFV could be detected after inoculation onto FTA cards, the vaccine substrain 17D-204, WT strain Asibi, and PBS as a negative control were inoculated onto FTA cards and assayed for YFV RNA. The viruses were inoculated at the same titer (6log_10_ pfu/mL). These C_t_ values were compared to a standard extraction (140 μL of sample directly extracted) of both 17D-204 and Asibi. Although the mean C_t_ values were lower when RNA was extracted from an FTA punch, both strain 17D-204 (mean C_t_ value: 23.2 ± 0.12) and strain Asibi (mean C_t_ value: 23.9 ± 0.1) were readily detected from FTA punches whereas the PBS control was negative for YFV RNA (mean Ct value: 43.3 ± 1.9) (S1 Fig).

### The low volume applied to FTA cards decreases assay sensitivity

Typically, when extracting viral RNA from a YFV clinical sample, 140 μL of serum is used as input in the extraction protocol. Although this volume can be applied to the entire FTA card, RNA extraction protocols utilizing FTA cards require the card to be punched prior to RNA extraction, limiting the volume of sample that is extracted. The timing of punching, pre-inoculation or post-inoculation was tested to ensure that the ‘pre-punch protocol’ did not impact RNA yield. There was no significant difference in C_t_ values when sample was applied to the whole card and punched later (“post-inoculation punch”, R^2^ = 0.99) and when sample was applied to pre-punched cards (“pre-inoculation punch”, R^2^ = 0.98) (pre-inoculation punch vs post-inoculation punch 95% CI: -9.3, 10.97, S2 Fig).

**Fig 1:**
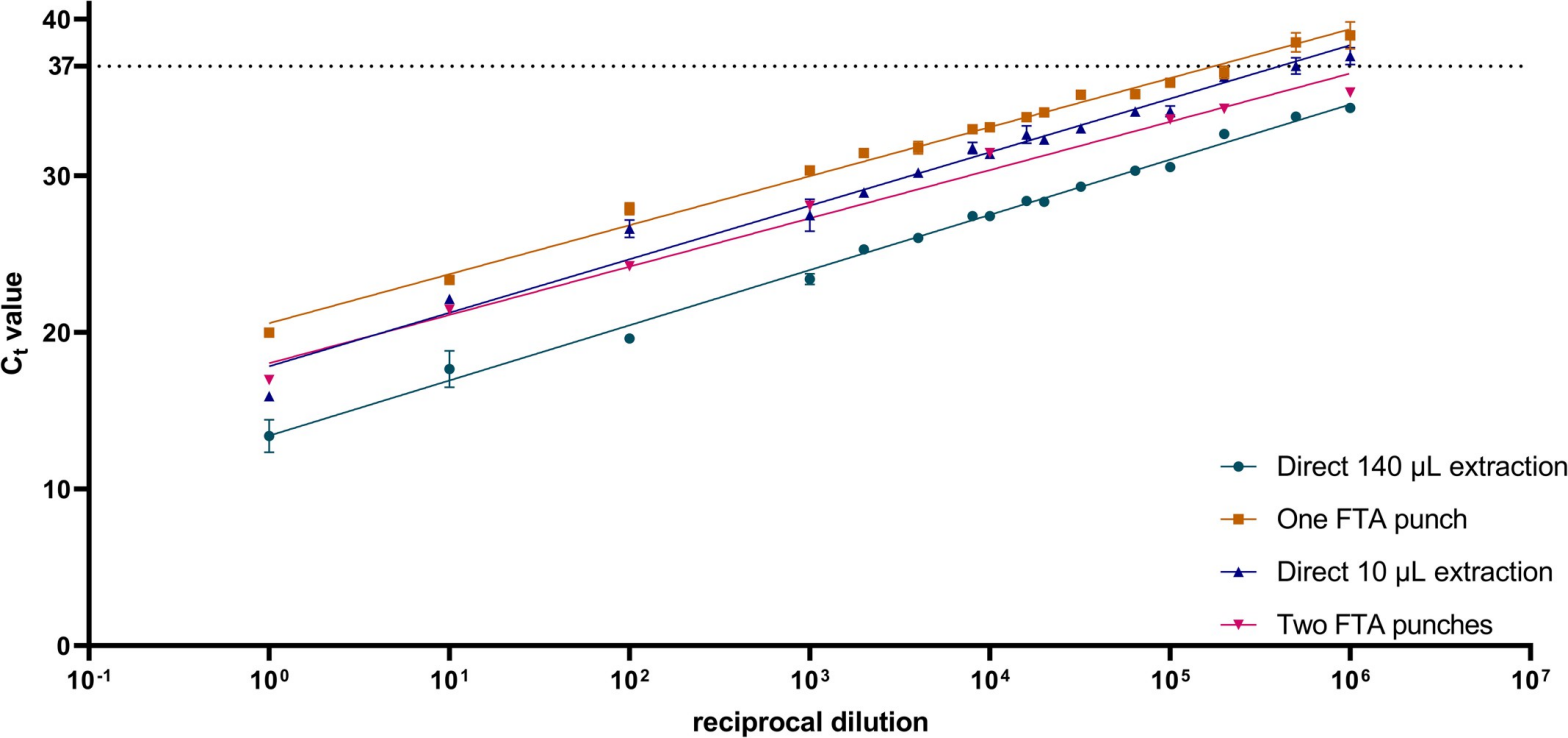
YFV RNA extraction from FTA punches lowers the sensitivity of YFV qRT-PCR assays based on volume, not technique. RNA from the vaccine strain of YFV, 17D-204, was extracted using the manufacturers protocol from 140 μL of YFV diluted in PBS, from a single FTA punch, from 10 μL of YFV diluted in PBS, and from two FTA punches. The dotted line at C_t_ value 37 indicates the cut off for YFV RNA positivity and is based on the cut-off used in the molecular diagnosis of YFV in the field. Dilutions to make the standard curve were performed in PBS. Points represent the average of two experiments and were fit using a linear regression.

To test if this reduction of input volume significantly impacts the sensitivity of the assay, the limit of detection of 10 μL virus applied to FTA punches was assayed and compared to a direct extraction from 10 μL of virus and the ‘gold standard’ of a direct extraction of 140 μL of virus. The LOD of 140 μL of virus (0.22 pfu, R^2^ = 0.98) was shown to be significantly lower (140 μL virus vs one FTA punch 95% CI: 0.09, 1.4) than the LOD of one FTA punch (3.01 pfu, R^2^ = 0.97); however, there was no significant difference (one FTA punch vs. 10 μL 95% CI: -0.2, 0.4) in the LOD of RNA extracted from one FTA punch compared to 10 μL of virus extracted directly (2.2 pfu, R^2^ = 0.97) (Fig 1). To determine if extracting RNA from multiple punches increased sensitivity, RNA was pooled from two punches inoculated with virus diluted in PBS (Fig 1). Increasing the number of punches utilized, significantly lowered (two FTA punch vs one FTA punch 95% CI: 0.4, 1.0) the LOD (0.6 pfu, R^2^ = 0.98) when compared to extracting RNA from one punch. This trend continued when RNA was extracted from three and four punches (S1 Table).

**Fig 2:**
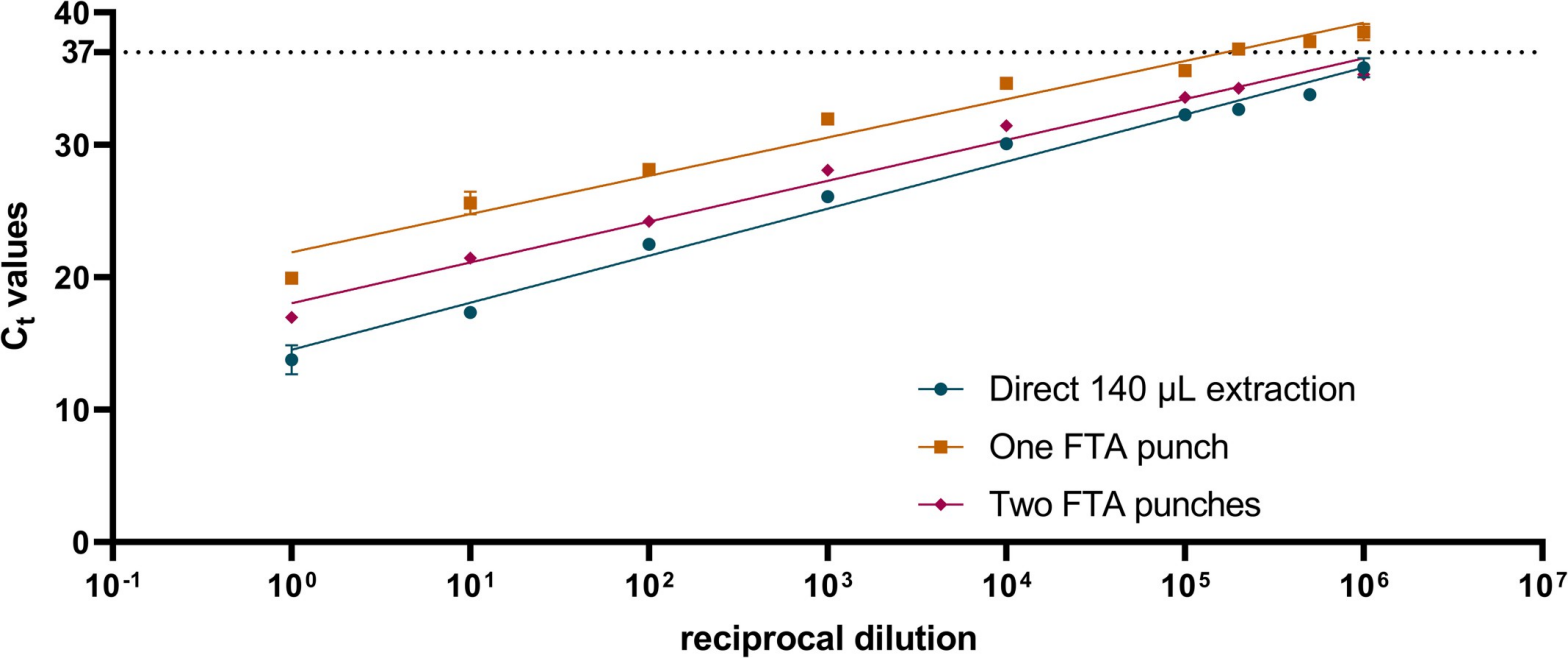
YFV spiked into serum can be extracted from FTA cards but with lower sensitivity than traditional methods. RNA from the vaccine strain of YFV, 17D-204, was extracted using the manufacturers protocol from 140 μL of YFV diluted in serum, from a single FTA punch, and from two FTA punches. The dotted line at C_t_ value 37 indicates the cut-off for YFV RNA positivity and is based on cutoffs used in the molecular diagnosis of YFV in the field. Dilution to make the standard curve were performed in human, flavivirus negative serum. Points represent the average of two experiments and were fit using a linear regression.

Because serum is the most common sample used for molecular diagnoses, the experiment was repeated using human, flavivirus-negative serum (EMD Millipore, Burlington, MA) as the diluent. As was observed in the experiment utilizing PBS as the diluent, the LOD of 140 μL of virus diluted in human serum (0.5 pfu, R^2^ = 0.96) was significantly lower (140 μL vs FTA punch 95% CI: 0.8, 1.5) than the LOD of an FTA punch inoculated with 10 μL virus diluted in human serum (2.7 pfu, R^2^ = 0.95), but the LOD of an FTA punch inoculated with 10 μL virus diluted in serum was not significantly different (10 μL serum vs 10 μL PBS 95% CI: -0.3, 0.4) than a FTA punch inoculated with 10 μL virus diluted in PBS (Fig 2).

### Heat decreased the sensitivity of YFV molecular diagnostics of FTA cards

**Fig 3:**
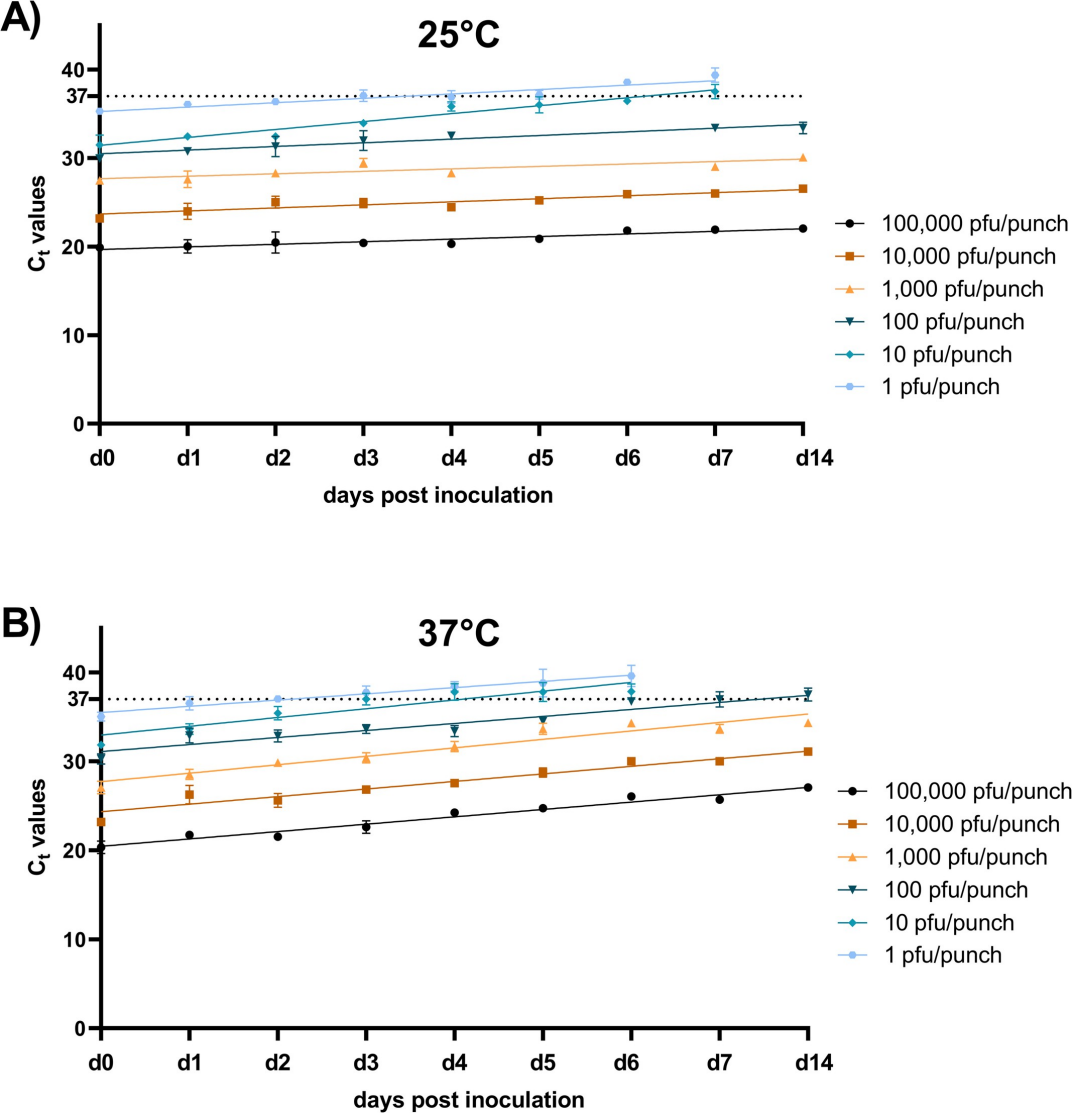
FTA card stabilization of YFV RNA decrease at high temperature. The vaccine strain of YFV, 17D-204, was diluted in PBS, spotted onto punches from FTA cards and placed at RT (~25°C) or 37°C for two weeks. The dotted line at C_t_ value 37 indicates the cut off for YFV RNA positivity and is based on cut-offs used in the molecular diagnosis of YFV in the field. Points represent the average of two experiments where extracts from one punch were made and were fit using a linear regression.

At room temperature, 100,000 pfu/punch (R^2^ = 0.68), 10,000 pfu/punch (R^2^ = 0.80), 1,000 pfu/punch (R^2^ = 0.64) and 100 pfu/punch (R^2^ = 0.82) could be detected for over two weeks (Fig 3A). YFV inoculated at 10 pfu/punch could be detected for 6.2 days (R^2^ = 0.88) and 1 pfu/punch for 3.5 days (R^2^ = 0.74) (Fig 3A). At 37°C, 100,000 pfu/punch (R^2^ = 0.78) and 10,000 pfu/punch (R^2^ = 0.73) were still detectable for more than 14 days; however, the 1,000 pfu/punch sample (R^2^ = 0.64) was detectable for 13.7 days (Fig 3A). Similarly, the duration of detectability for the 100 pfu/punch (10.1 days, R^2^ = 0.70), 10 pfu/punch (4.1 days, R^2^ = 0.77) and 1 pfu/punch (2.2 days, R^2^ = 0.72) samples decreased at 37°C (Fig 3B). Using 95% confidence intervals to compare the number of days YFV was detectable by qRT-PCR, 10 pfu/punch (RT vs 37°C 95% CI: 1.52, 2.77) and 1 pfu/punch (RT vs 37°C 95% CI: 0.7, 2.0) became undetectable in a significantly shorter time at 37°C than at RT.

To test if RNA extraction from multiple FTA punches improved the sensitivity of YFV molecular detection at high temperatures, RNA was pooled from two punches at 10 pfu/punch and 1 pfu/punch. It was shown that extracting RNA from two punches significantly increased the number of days RNA could be detected (Table 1).

**Table 1:** Increased temperature decreases the duration of YFV RNA positivity when extracted from FTA cards.

		Single punch		Double punch		95% confidence interval
	Titer	Days*	R^2^**	Days*	R^2^**	
RT	10 pfu/punch	6.2	0.68	9.2	0.85	(2.12,4.11)
	1 pfu/punch	3.5	0.8	6.6	0.81	(2.40,4.02)
37°C	10 pfu/punch	4.1	0.76	6.0	0.77	(1.13,2.77)
	1 pfu/punch	2.2	0.72	3.7	0.87	(1.06,2.14)

*Days refers to the number of days YFV RNA was detected by qRT-PCR and was calculated by averaging the results of three replicates.

**R^2^ values were calculated by performing a linear regression using qRT-PCR data over one week.

### High humidity negatively impacts RNA stability on FTA cards

The initial experiments were conducted in a controlled climate (40–55% humidity). To test if these humidity conditions positively influenced RNA stability on the FTA cards, cards were inoculated with 10 pfu/punch and 1 pfu/punch of YFV and placed into a humidity chamber (80–85%) for one week. Control samples were left in the biosafety cabinet at ambient humidity for one week. Cards incubated at high humidity showed no difference in the time limit for detection of positive C_t_ values when compared to dry cards incubated at low humidity (S2 Table). Despite this, the negative effect of humidity on the ability of FTA cards to stabilize RNA has been frequently documented in regions endemic to YFV where high humidity is common [28]. To assay if the reported loss in RNA yield was due to the card having absorbed moisture from the air prior to inoculation, cards were pre-incubated in the humidity chamber for one, two or three days before inoculation with YFV (illustrated in S3 Fig). As the pre-incubation period increased, the LOD decreased significantly (S3 Table, 1 pfu/punch low humidity vs 1 pfu/punch high humidity 3 days 95% CI: -4.78, -2.83)). The difference in LOD between one and two days of preincubation was not significant (one day vs. two days 95% CI: -0.1, 0.7); however, the difference in LOD after three days was significantly different (two days vs. three days 95% CI: 0.1, 0.9). At 10 pfu/punch, there was no difference in the period of detection between one and two days (one day vs. two days 95% CI: -0.6, 1.5) but there was a significant difference between two and three days (two days vs. three days 95% CI: 2.4, 4.7) (Table 2). There was a significant difference in time of detection at 1 pfu/punch between both one and two (one day vs. two days 95% CI: 0.2, 3.0) and two and three days (two days vs. three days 95% CI: 2.8, 5.1) of preincubation in high humidity. YFV RNA was undetectable at 1 pfu/punch after preincubating cards for three days in high humidity (Table 2).

**Table 2:** Storage of FTA cards in humid environments negatively impacts the ability of the cards to stabilize YFV RNA over time.

			FTA cards were pre-incubated at high humidity					
	low humidity		one day		two days		three days	
Titer	Days*	R^2^**	Days*	R^2^**	Days*	R^2^**	Days*	R^2^**
100,000 pfu/punch	128.7	0.68	28.4	0.97	26.8	0.88	16.5	0.87
10,000 pfu/punch	67.3	0.80	16.8	0.91	14.5	0.84	10.5	0.84
1,000 pfu/punch	47.8	0.82	15.9	0.86	8.0	0.96	7.2	0.90
100 pfu/punch	27.4	0.79	12.2	0.82	6.4	0.93	5.1	0.80
10 pfu/punch	6.2	0.88	3.6	0.62	3.3	0.71	2.8	0.87
1 pfu/punch	3.5	0.74	3.4	0.64	2.2	0.40	-	-

*Days refers to the number of days YFV RNA was detected by qRT-PCR and was calculated by averaging the results of three replicates.

**R^2^ values were calculated by performing a linear regression using qRT-PCR data over one week.

### Desiccating FTA cards prior to inoculation improves YFV RNA stability

In an attempt to reduce the loss of RNA yield at high humidity, pre-humidified cards were incubated with desiccation packets for one, two and seven days prior to inoculation with low titer YFV (illustrated in S3 Fig). The addition of desiccation packets resulted in an extended detection time compared to the same concentration/punch incubated with no desiccation (Table 3). For punches inoculated with 10 pfu/punch, two desiccation packets significantly extended the length of detection after one day of desiccation (95% CI: 0.1, 1.1) compared to one packet. For punches inoculated with 1 pfu/punch, there was not a significant change in the length of detection between cards pre-incubated for one or two days with one or two desiccant packets. For cards incubated for 7 days prior to inoculation with 10 pfu/punch (one desiccation packet vs two desiccation packets 95% CI: 0.2, 2.5) and 1 pfu/punch (one desiccation packet vs two desiccation packets 95% CI: 1.4, 3.4) of YFV, the addition of two desiccation packets significantly extended RNA detection when compared to the addition of one packet (Table 3). When compared to FTA punches incubated at low humidity (Table 1), one or two days of desiccation prior to inoculation resulted in similar RNA stability (Table 2). Two brands of desiccant packets were tested and after two days of desiccation, there was no significant difference in the length of detection for either titer of YFV tested (S4 Table).

**Table 3:** FTA card desiccation prior to inoculation improves YFV RNA stabilization.

		no desiccation packets		one desiccation packet		two desiccation packets		95% confidence interval^+^
Incubation in high humidity	Titer	Days*	R^2^**	Days*	R^2^**	Days*	R^2^**	
1 day	*10 pfu/punch*	3.8	0.86	6.3	0.91	6.9	0.96	(0.1,1.1)
2 days		3.80	0.73	6.3	0.86	6.3	0.88	(-0.8,0.8)
7 days		3.3	0.74	4.8	0.93	5.9	0.73	(0.2,2.5)
1 day	*1 pfu/punch*	1.8	0.66	4.5	0.9	4.3	0.97	(-0.2,0.6)
2 days		1.6	0.32	4.5	0.9	4.5	0.85	(-0.5,0.7)
7 days		-	-	2.1	0.59	4.4	0.64	(1.4,3.4)

*Days refers to the number of days YFV RNA was detected by qRT-PCR

**R^2^ values were calculated by performing a linear regression using qRT-PCR data over one week.

^+^Confidence interval reported compares the 95% confidence intervals of one desiccation packet to the 95% confidence interval of two desiccation packets.

- Indicates that YFV RNA was unable to be detected after one day

## Discussion

The molecular diagnosis of YFV can be limited by the rate at which viral RNA degrades, which is accelerated by the hot and humid conditions present in many endemic regions [29]. Because RNA is highly labile, suspected YFV clinical samples must be transported using continuous cold-chain maintenance, with samples arriving within a day of departure being shipped on wet ice, and those in transit for more than one day being shipped at -20°C [3]. Whatman FTA cards have been employed as a solution for other RNA viruses as they rapidly inactivate virus and stabilize genetic material [11,24,30–32]. Protocols utilizing a wide variety of sample types have been optimized using FTA cards including blood, serum, tick and mosquito homogenates, dead bird impressions, throat swabs, oral fluid and epithelial suspensions [13,18,22–24,30–36]. In fact, the WHO and CDC approved the use of FTA cards for measles and poliovirus diagnostic protocols, indicating that the cards are effective in a clinical setting [23]. In this study we optimize the use of Whatman FTA cards in conjunction with YFV molecular diagnostics and show that the incorporation of this widely available commercial product could lessen the requirement for cold-chain, which is often unavailable or incomplete.

During a YFV outbreak, molecular diagnostics are highly important as IgM generated in response to WT YFV infection and vaccination are indistinguishable via serology. In this study, the YFall qRT-PCR assay [9] was used to show that WT and vaccine strains of YFV were equally detectable after inoculation onto FTA cards (S1 Fig). This molecular assay is commonly used in the field and has been shown to be both sensitive and specific for WT and vaccine strains of YFV [8,9]. We have previously designed an qRT-PCR assay that distinguishes between the two strains using locked nucleic acid (LNA) technology and believe that the use of FTA cards to transport clinical samples during an outbreak could increase the successful use of both molecular assays.

Viremia/RNA in sera during WT YFV infection is transient, especially in mild or subclinical cases, and usually begins to wane 3–6 days after infection [1]. Viremia can last the duration of infection in severe cases of YF but is highly variable [37,38]. In a study of the 2016–2018 Brazilian YFV outbreak viremia at time of death ranged from over 10^5^ pfu/mL to less than 1 pfu/mL [39]. In many cases, the sensitivity of molecular assays was shown to decrease with the use of FTA cards [23,28,31]. It is hypothesized that this is due to many factors, including the volume of inoculum used, the humidity of endemic regions and incomplete drying of FTA card post-inoculation with sample [23,28,40]. Although the LOD of YFV 17D RNA on FTA punches was shown to be less than 10 pfu/mL in both PBS and flavivirus-negative human sera, the LOD of FTA cards was significantly different than the gold standard of direct RNA extraction. Although achieving 100% of RNA recovery in the extraction is unlikely, loss in RNA yield was likely due to the volume of sample used to extract RNA as the FTA punch holds only 10 μL whereas a direct extraction involves 140 μL of clinical specimen. By extracting RNA from two or more punches, the LOD was significantly improved. Up to nine, 6 mm punches can be made from FTA Micro cards, which means RNA from multiple punches can be pooled leaving enough card for confirmatory replicates to be easily achieved. The reduction in sensitivity when using FTA cards compared to direct RNA extraction may be overcome by the use of multiple punches to allow detection of less than 1 pfu/mL. This would allow for detection of the full range of viral loads seen in YF clinical samples.

The most common sample type used in molecular diagnosis of YF is serum. Using 17D-204 virus spiked into flavivirus-negative human serum, we showed no significant difference in RNA preservation compared to 17D-204 virus diluted in PBS. Many studies have shown flavivirus RNA may be more readily detected and detected for longer periods of time in whole blood, as the virus binds strongly to red blood cells [41–43]. Though the effect of whole blood on YFV molecular diagnostics was not tested here, molecular diagnosis has been made from whole blood placed on FTA cards for Anaplasma and Rickettsia species [15,16,44]. Taken together, it is likely that blood samples taken from suspected YF patients would be another acceptable sample type for YF molecular diagnosis from FTA cards. The ability to submit finger prick blood draws could increase the ease of YFV surveillance in remote regions where cold transport is unavailable.

YFV transmission season coincides with the rainy season in both South American and African regions, which also corresponds to the hottest time of the year. FTA cards have been shown to stabilize RNA at high temperature, although the duration of stabilization does decrease as temperature increases [22]. Here, at 37°C, high titer YFV RNA was preserved on FTA cards for more than two weeks and low titer RNA was preserved for two days at 37°C. Although there was a reduction in the time YFV RNA could be detected at high temperature compared to room temperature, two days is within the WHO’s current timeline for testing of YFV samples. This suggests that low titer samples would be detected after transport in normal conditions; however, during outbreak settings transport can exceed two days. Extracting RNA from two punches improved the time for RNA to be detectable to 3.7 days, further illustrating the utility of the double punch method. Extreme environmental conditions in endemic regions (i.e., higher temperatures) were not tested here, which is a limitation of this study. However, field trials will be done in different countries to analyze their impact on the YF RNA stabilization on FTA cards.

The high humidity of transmission season is also proposed to limit RNA stabilization by FTA cards [28]. One study showed that incomplete drying of FTA cards caused the most significant loss of RNA yield [23]. Our data confirms this finding as storing cards for three days at high humidity affected RNA preservation more than three days of high temperature. To discern how to best mitigate this loss in RNA, the impact of humidity before and after inoculation was tested. Cards that were inoculated dry and then placed at high humidity showed no significant loss in yield; however, cards exposed to a highly humid environment for days prior to inoculation (Table 2), displayed a significant loss in RNA detection. This suggests that high humidity decreases the ability of FTA cards absorb inoculum as water is absorbed from the environment by the FTA card, limiting sample capacity as others have noted [22,23,28,32]. To counteract absorption of environmental humidity, ‘pre-humidified’ FTA cards were desiccated prior to, and after inoculation. The data presented here showed that two days of pre-desiccation significantly improved RNA stabilization. Desiccating FTA cards for longer was shown to be less effective, which is likely due to desiccation packets becoming fully saturated. If desiccation packets had been swapped for new packets, RNA may have been better stabilized. Nevertheless, YFV inoculated onto pre-desiccated cards could be detected days longer than cards that had been stored in humid conditions. This suggests that if cards are dry when samples are applied and stored in sealed containers with desiccation packets, RNA can be successfully stabilized and detected.

FTA cards have been successfully incorporated into the molecular diagnostic protocols for many RNA viruses. Here we showed that FTA cards stabilized and inactivated YFV in average environmental conditions. The implementation of FTA cards could aid YFV surveillance in remote regions by simplifying and ensuring success of diagnostically-viable sample transport to laboratories. The use of FTA cards could make the difference between complete loss of detectable RNA and successful detection in many instances. Early detection of YF cases is integral to the EYE strategy for controlling YF outbreaks [3]. The ability to reliably detect YFV RNA would reduce the burden on serological testing which is often complicated in endemic regions and during an outbreak scenario. As the molecular diagnosis of YFV becomes more routine in YF-endemic regions, RNA stabilization provided by FTA cards may become integral to ensuring accurate diagnosis.

## Supporting information

S1 Table Increasing the number of punches used in RNA extraction, improves detection. (DOCX)

S2 Table Pre-desiccated FTA cards perform similarly at low and high humidity. (DOCX)

S3 Table Incubating FTA cards at high humidity prior to inoculation decreases LOD of YFV RNA extracted from cards. (DOCX)

S4 Table Brand of 100g desiccation packet does not affect length of YFV RNA detection. (DOCX)

S1 Fig WT and vaccine strains of YFV can be detected using FTA cards. WT YFV strain Asibi and YFV vaccine strain 17D-204 were spotted onto FTA cards and allowed to dry for one hour. RNA was extracted from the cards and assayed using YFall qRT-PCR primers. RNA was also extracted directly from the Asibi and 17D-204 isolates using 140 μL of sample directly into lysis buffer. PBS was spotted onto FTA cards as a negative control. The dotted line at C_t_ value 37 indicates the cut-off for YFV RNA positivity and is based on cut-offs used in the molecular diagnosis of YFV in the field.
(TIF)

S2 Fig Punching FTA cards pre- or post-inoculation of sample does not impact RNA yield. YFV 17D-204 virus was inoculated onto FTA cards (130 μL sample over entire card) or onto FTA card punches (10 μL/ punch) and allowed to dry completely. RNA was then extracted from punches made from the whole FTA card or the pre-punched samples. The dotted line at C_t_ value 37 indicates the cut-off for YFV RNA positivity and is based on cut-offs used in the molecular diagnosis of YFV in the field. Dilutions were performed in PBS. Points and error bars represent the average of two experiments and data was fit using a linear regression.
(TIF)

S3 Fig Experimental design of effects of humidity YFV RNA yield from FTA cards. Made using BioRender.
(PNG)
