# Correction: Evaluation of Whatman FTA cards for the preservation of yellow fever virus RNA for use in molecular diagnostics

**DOI:** 10.1371/journal.pntd.0011027

**Published:** 2022-12-30

**Authors:** Emily H. Davis, Jason O. Velez, Brandy J. Russell, A. Jane Basile, Aaron C. Brault, Holly R. Hughes

This manuscript is missing an Acknowledgements section. The Acknowledgements should include the following statement: The findings and conclusions in this report are those of the authors and do not necessarily represent the official position of the Centers for Disease Control and Prevention (CDC).
