# Supplementary material for: Evaluation of Whatman FTA cards for the preservation of yellow fever virus RNA for use in molecular diagnostics
Source: PLoS Negl Trop Dis. 2022 Jun 15;16(6):e0010487. doi: 10.1371/journal.pntd.0010487 (PMC9200311; doi:10.1371/journal.pntd.0010487)
Supplement: S1 Table — (DOCX) [file pntd.0010487.s001.docx]

**S1 Table: Increasing the number of punches used in RNA extraction, improves detection**

|  | 10 pfu/punch | | 1 pfu/punch | |
| --- | --- | --- | --- | --- |
|  | Mean C_t_ | Std Dev | Mean C_t_ | Std Dev |
| 40 µL | 30.6 | 0.3 | 32.4 | 0.2 |
| 4 punches | 30.9 | 0.08 | 34.6 | 0.3 |
| 30 µL | 31 | 0.2 | 34.4 | 0.3 |
| 3 punches | 31.1 | 0.1 | 34.6 | 0.3 |
| 20 µL | 31.5 | 0.3 | 34.2 | 0.2 |
| 2 punches | 31.7 | 0.5 | 35.1 | 0.2 |
| 10 µL | 32.1 | 0.2 | 35.3 | 0.2 |
| 1 punch | 32.5 | 0.3 | 35.5 | 0.1 |
