# Supplementary material for: Evaluation of Whatman FTA cards for the preservation of yellow fever virus RNA for use in molecular diagnostics
Source: PLoS Negl Trop Dis. 2022 Jun 15;16(6):e0010487. doi: 10.1371/journal.pntd.0010487 (PMC9200311; doi:10.1371/journal.pntd.0010487)
Supplement: S2 Table — (DOCX) [file pntd.0010487.s002.docx]

**S2 Table: Pre-desiccated FTA cards perform similarly at low and high humidity**

|  | low humidity | | high humidity | | Difference (95% confidence interval) |
| --- | --- | --- | --- | --- | --- |
| Titer | Days* | R^2**^ | Days* | R^2**^ |  |
| 10 pfu/punch | 6.6 | 0.92 | 6.9 | 0.9 | (-0.3,0.9) |
| 1 pfu/punch | 3.6 | 0.51 | 3.3 | 0.71 | (-0.7,2.1) |

*Days refers to the number of days YFV RNA was detected by qRT-PCR

**R^2^ values were calculated by performing a linear regression using qRT-PCR data over one week.
