# Supplementary material for: Evaluation of Whatman FTA cards for the preservation of yellow fever virus RNA for use in molecular diagnostics
Source: PLoS Negl Trop Dis. 2022 Jun 15;16(6):e0010487. doi: 10.1371/journal.pntd.0010487 (PMC9200311; doi:10.1371/journal.pntd.0010487)
Supplement: S3 Table — (DOCX) [file pntd.0010487.s003.docx]

**S3 Table: Incubating FTA cards at high humidity prior to inoculation decreases LOD of YFV RNA extracted from cards**

| Condition | LOD (days) | R^2*^ |
| --- | --- | --- |
| 24-hour pre-incubation | 15.4 | 0.97 |
| 48-hour pre-incubation | 15.8 | 0.95 |
| 96-hour pre-incubation | 16.6 | 0.97 |

**R^2^ values were calculated by performing a linear regression using qRT-PCR data over one week.
