# Supplementary material for: Evaluation of Whatman FTA cards for the preservation of yellow fever virus RNA for use in molecular diagnostics
Source: PLoS Negl Trop Dis. 2022 Jun 15;16(6):e0010487. doi: 10.1371/journal.pntd.0010487 (PMC9200311; doi:10.1371/journal.pntd.0010487)
Supplement: S4 Table — (DOCX) [file pntd.0010487.s004.docx]

**S4 Table: Brand of 100g desiccation packet does not affect length of YFV RNA detection**

|  |  | Dry & dry brand | | FTA brand | | Difference (95% confidence interval) |  |
| --- | --- | --- | --- | --- | --- | --- | --- |
|  |  | Days* | R^2**^ | Days* | R^2**^ |  |  |
| 10 pfu/punch | *no packets* | 3.8 | 0.73 | 3.15 | 0.87 | (-0.1,1.2) |  |
|  |  |  |  |  |  |  |  |
|  | *1 packet* | 6.26 | 0.86 | 5.88 | 0.84 | (-0.6,1.5) |  |
|  |  |  |  |  |  |  |  |
|  | *2 packets* | 6.32 | 0.88 | 5.47 | 0.91 | (-0.2,1.6) |  |
|  |  |  |  |  |  |  |  |
| 1 pfu/punch | *no packets* | 1.56 | 0.32 | 1.36 | 0.66 | (-1.4,1.5) |  |
|  |  |  |  |  |  |  |  |
|  | *1 packet* | 4.46 | 0.9 | 4.37 | 0.9 | (-0.5,0.8) |  |
|  |  |  |  |  |  |  |  |
|  | *2 packets* | 4.53 | 0.85 | 5.09 | 0.92 | (-0.1,1.2) |  |
|  |  |  |  |  |  |  |  |

**R^2^ values were calculated by performing a linear regression using qRT-PCR data over one week.
