# Supplementary figures and images for: Evaluation of Whatman FTA cards for the preservation of yellow fever virus RNA for use in molecular diagnostics
Source: PLoS Negl Trop Dis. 2022 Jun 15;16(6):e0010487. doi: 10.1371/journal.pntd.0010487 (PMC9200311; doi:10.1371/journal.pntd.0010487)

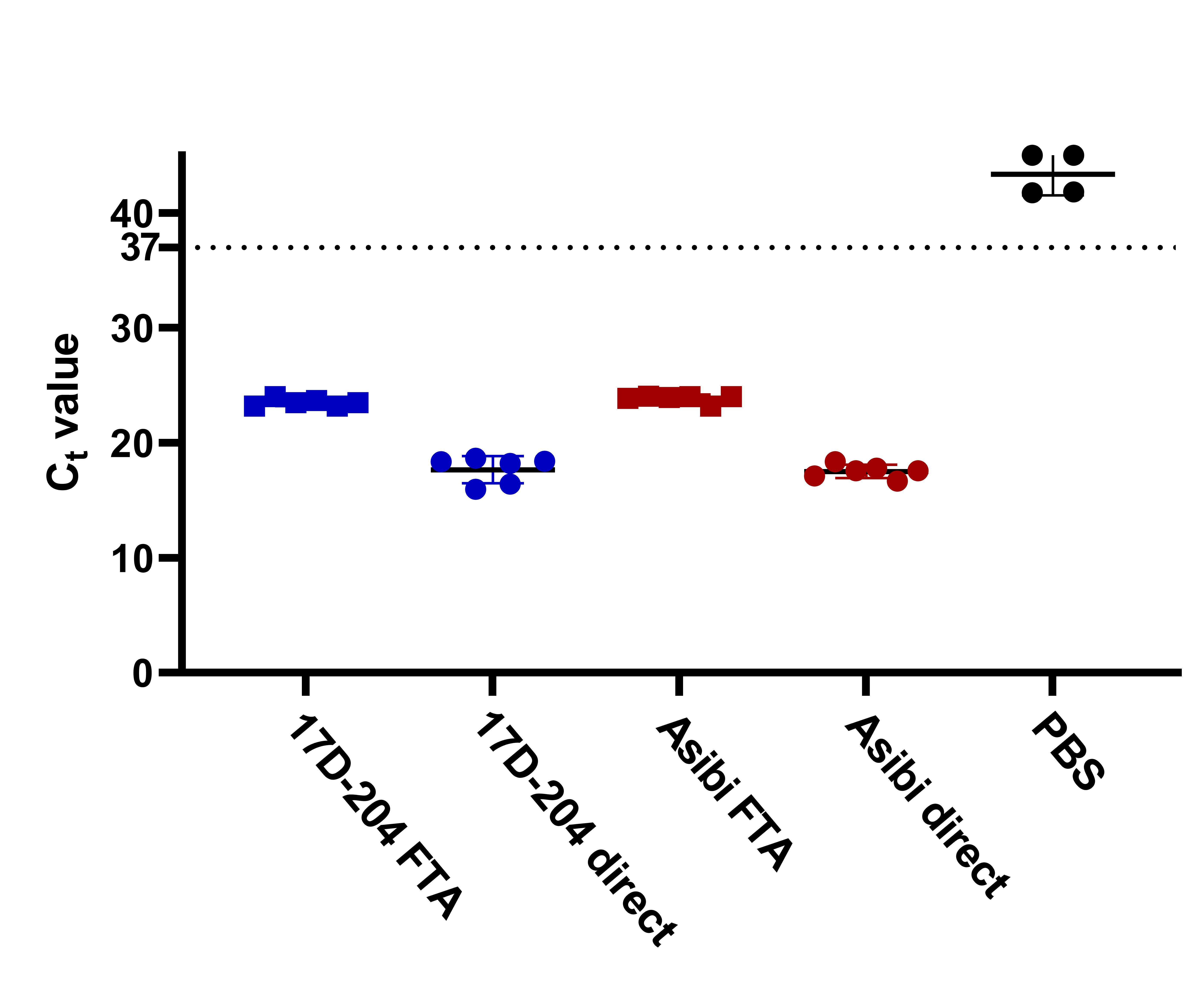

Supplement: S1 Fig — WT YFV strain Asibi and YFV vaccine strain 17D-204 were spotted onto FTA cards and allowed to dry for one hour. RNA was extracted from the cards and assayed using YFall qRT-PCR primers. RNA was also extracted directly from the Asibi and 17D-204 isolates using 140 μL of sample directly into lysis buffer. PBS was spotted onto FTA cards as a negative control. The dotted line at Ct value 37 indicates the cut-off for YFV RNA positivity and is based on cut-offs used in the molecular diagnosis of YFV in the field. (TIF) [file pntd.0010487.s005.tif]

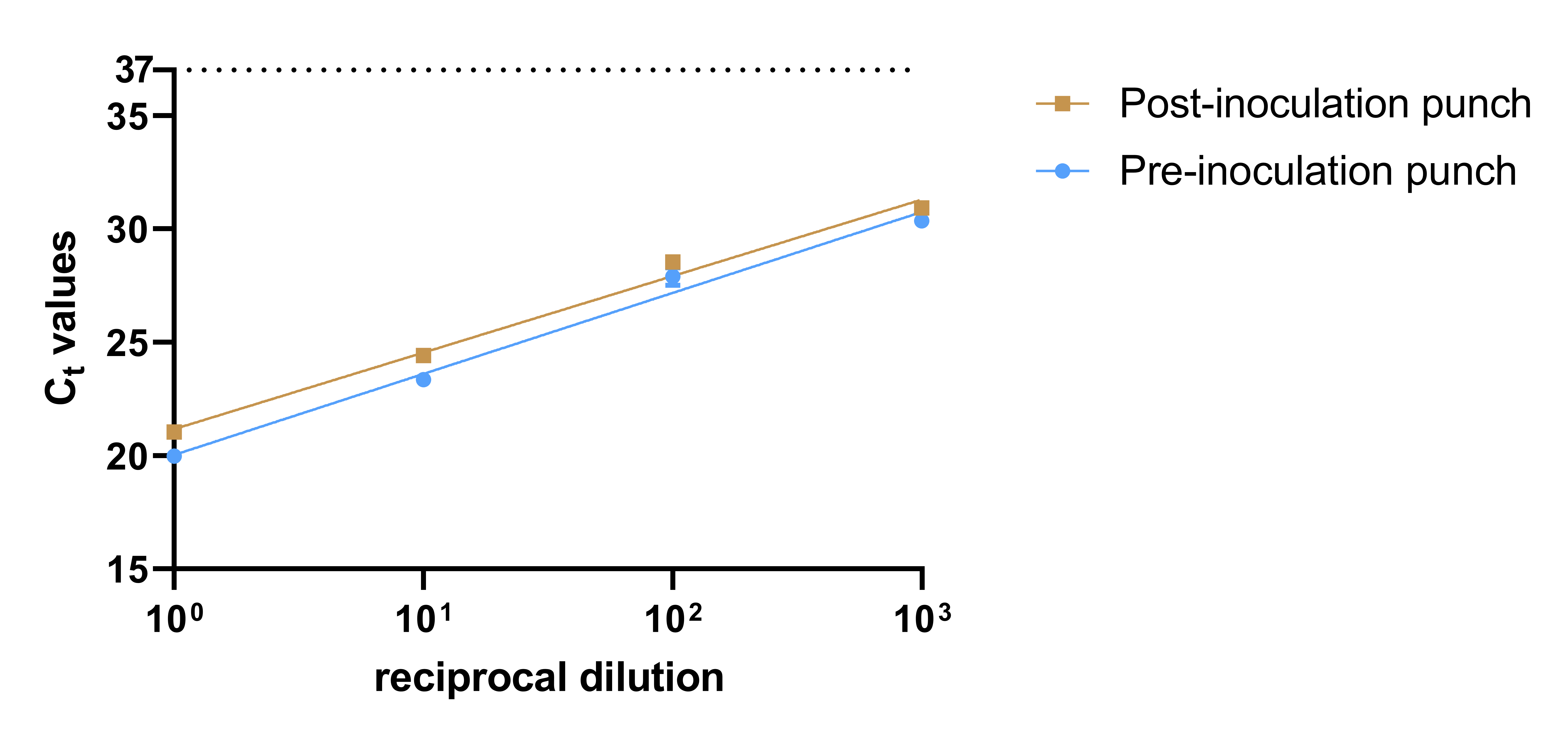

Supplement: S2 Fig — YFV 17D-204 virus was inoculated onto FTA cards (130 μL sample over entire card) or onto FTA card punches (10 μL/ punch) and allowed to dry completely. RNA was then extracted from punches made from the whole FTA card or the pre-punched samples. The dotted line at Ct value 37 indicates the cut-off for YFV RNA positivity and is based on cut-offs used in the molecular diagnosis of YFV in the field. Dilutions were performed in PBS. Points and error bars represent the average of two experiments and data was fit using a linear regression. (TIF) [file pntd.0010487.s006.tif]

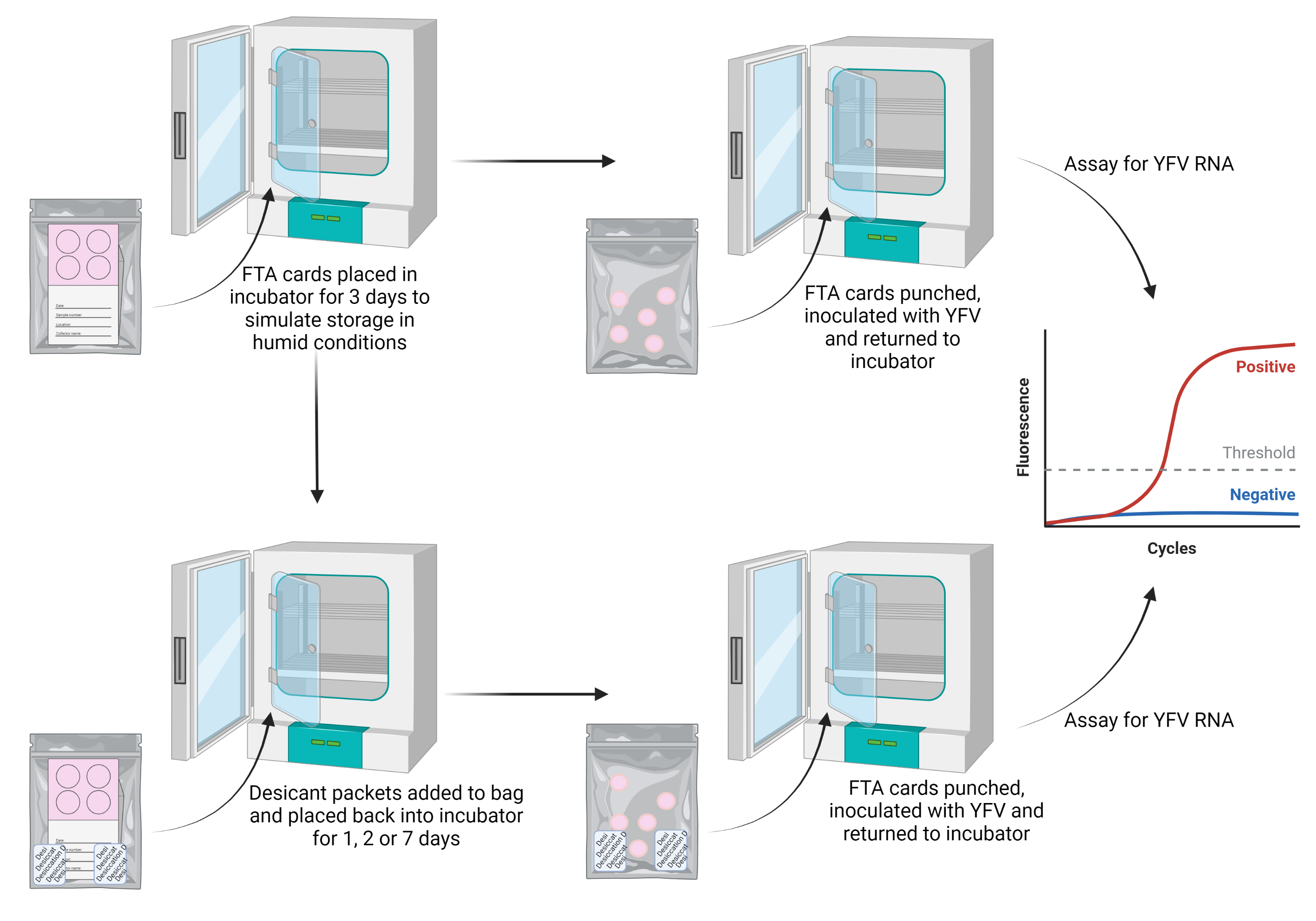

Supplement: S3 Fig — Made using BioRender. (PNG) [file pntd.0010487.s007.png]
